# T Cell Receptor-Like Recognition of Tumor *In Vivo* by Synthetic Antibody Fragment

**DOI:** 10.1371/journal.pone.0043746

**Published:** 2012-08-20

**Authors:** Keith R. Miller, Akiko Koide, Brenda Leung, Jonathan Fitzsimmons, Bryan Yoder, Hong Yuan, Michael Jay, Sachdev S. Sidhu, Shohei Koide, Edward J. Collins

**Affiliations:** 1 Department of Biochemistry and Biophysics, University of North Carolina, Chapel Hill, North Carolina, United States of America; 2 Department of Biochemistry and Molecular Biophysics, University of Chicago, Chicago, Illinois, United States of America; 3 Department of Pharmaceutical Sciences, University of North Carolina, Chapel Hill, North Carolina, United States of America; 4 Department of Radiology, University of North Carolina, Chapel Hill, North Carolina, United States of America; 5 Donnelly Centre, University of Toronto, Toronto, Ontario, Canada; 6 Department of Microbiology and Immunology, University of North Carolina, Chapel Hill, North Carolina, United States of America; Genentech, United States of America

## Abstract

A major difficulty in treating cancer is the inability to differentiate between normal and tumor cells. The immune system differentiates tumor from normal cells by T cell receptor (TCR) binding of tumor-associated peptides bound to Major Histocompatibility Complex (pMHC) molecules. The peptides, derived from the tumor-specific proteins, are presented by MHC proteins, which then serve as cancer markers. The TCR is a difficult protein to use as a recombinant protein because of production issues and has poor affinity for pMHC; therefore, it is not a good choice for use as a tumor identifier outside of the immune system. We constructed a synthetic antibody-fragment (Fab) library in the phage-display format and isolated antibody-fragments that bind pMHC with high affinity and specificity. One Fab, fE75, recognizes our model cancer marker, the Human Epidermal growth factor Receptor 2 (HER2/neu) peptide, E75, bound to the MHC called Human Leukocyte Antigen-A2 (HLA-A2), with nanomolar affinity. The fE75 bound selectively to E75/HLA-A2 positive cancer cell lines *in vitro*. The fE75 Fab conjugated with ^64^Cu selectively accumulated in E75/HLA-A2 positive tumors and not in E75/HLA-A2 negative tumors in an HLA-A2 transgenic mouse as probed using positron emission tomography/computed tomography (PET/CT) imaging. Considering that hundreds to thousands of different peptides bound to HLA-A2 are present on the surface of each cell, the fact that fE75 arrives at the tumor at all shows extraordinary specificity. These antibody fragments have great potential for diagnosis and targeted drug delivery in cancer.

## Introduction

One of the most important lessons learned about cancer in the past 100 years is the understanding that each cancer is as unique as the patient. Effective treatment of the patient is linked to knowing which proteins, such as hormone receptors or HER2/neu, are actively expressed in the tumor. Probes that allow us to ascertain the molecular details of each patient's tumors will greatly enhance treatment. Biopsies can be used to determine molecular details, but biopsy is invasive, not always possible and does not allow for the fact that the tumor may change characteristics over time or location [1]. It would be preferable to be able to perform a noninvasive technique first followed by treatment [2]. Our long-term goal is to develop agents that can specifically target tumor cells for detection and delivery of toxins or chemotherapeutics.

Any noninvasive approach to determining molecular details of the tumor must use a probe that binds to the tumor cells with high affinity and high specificity. Monoclonal antibodies with affinity for cell-surface receptors on tumors have had great success in the clinic, but this approach appears to be limited because of the expression of that same cell surface receptor on normal cells that cause side effects during treatment. One of the best examples of successful use of monoclonal antibodies to tumor-associated antigens is Herceptin. Herceptin targets the human epidermal growth factor receptor 2 (HER2/neu). HER2/neu is over-expressed in 15–30% of solid tumors including breast, ovarian, colorectal, esophageal, squamous head and neck and stomach [3]. Over-expression of HER2/neu correlates with poor prognosis for therapy of breast cancer [4]–[8] and has been linked to increased metastases [9]. Monoclonal antibodies directed towards HER2/neu such as Herceptin have shown great efficacy in the clinic, but patients do develop resistance over time [10] and some patients do not respond at all [11]. Therefore, other approaches to target HER2/neu over-expressing tumor cells would be of significant value.

Our approach to targeting HER2/neu positive tumor cells is to use the same mechanism that the immune system uses to target and kill tumors, that is to target peptides derived from the HER2/neu protein when bound and presented by the Major Histocompatibility Complex (MHC). The interaction of the T cell receptor (TCR) with peptide/MHC complex is the fundamental event that triggers the adaptive immune response [12]. T cells identify tumor cells by recognizing peptides derived from tumor-associated molecules. The TCR would theoretically be an excellent probe to use for tumors, except that the TCR is frequently not amenable to manipulation and recombinant expression and its affinity for particular pMHC is relatively poor (the dissociation constant in the high μM range) [13]–[19]. Monoclonal antibodies would be a much better approach, because they are robust molecules and their production is well established. Antibodies that target specific pMHC on the surface of tumor cells have been produced but for some unknown reason, producing them conventionally is very difficult. When available, these TCR-like antibody fragments appear to bind similarly to TCR binding pMHC [20], [21]. Antibodies (or antibody fragments) previously isolated from phage-display libraries exhibited relatively low affinity for the pMHC complexes [22]–[24].

In this study, we isolated a T-cell receptor like Fab that specifically targets the HER2/neu peptide, E75 (KIFGSLAFL), bound to the human MHC, Human Leukocyte Antigen, HLA-A2 from a synthetic antibody library using phage display. The Fab binds with nanomolar binding affinity and high specificity for the E75/HLA-A2 pMHC molecule. The Fab was then modified to attach radioactive ^64^Cu and used for *in vivo* PET/CT imaging experiments on tumor-bearing mice that express HLA-A2 transgenically. The Fab showed increased retention in the HER2/neu pMHC positive tumors compared to HER2/neu pMHC negative tumors. These TCR-like antibodies can be used to study antigens presented on diseased and antigen-presenting cells and for delivering effective therapies in tumor and T-cell based diseases.

## Materials and Methods

### Ethics Statement

This study was completed in strict accordance with the Association for Assessment and Accreditation of Laboratory Animal Care at the University of North Carolina (UNC) Animal Facility and the mice were handled according to the UNC Office of Animal Care and Use. All experimentation was in accordance with the protocol (08–235.0) approved by the UNC Institutional Animal Care and Use Committee.

### Cells and Culture Conditions

MDA-MB-231, MCF7, and LNCAP tumor cells, CHO cells, T2 lymphoblast cells were purchased from ATCC. SKOV3 and SKOV3 cells transfected with HLA-A2 were a generous gift from Dr. Jonathan Serody (Department of Microbiology, UNC – Chapel Hill, NC) [25]. Unless otherwise mentioned all media supplies were purchased from Mediatec, Inc. (Manassas, VA). Each of the cell lines were grown in RMPI 1640 supplemented with 10% heat-inactivated FBS (Atlanta Biologicals, Inc.; Lawrenceville, GA), 10 mM sodium pyruvate, and penicillin/streptomyocin in humidified CO_2_ (5%) incubator at 37°C. SKOV3 transfected HLA-A2 cells and CHO transfected HLA-A2 cells were grown in the same conditions as above with the addition of 2.5 mg/ml G418.

### Antibodies and synthetic peptides

Anti-c-erb B-2 Ab-2 (9G6.10) was purchased from NeoMarkers (Fremont, Ca). Alexa Fluor 647 labeled Goat anti-mouse IgG1, PE labeled mouse IgG2b, and Alexa Fluor 647 labeled Streptavidin were purchased from Invitrogen, Inc. PE labeled mouse anti-Human HLA-A2 clone BB7.2 was purchased from BD Biosciences. The peptides: E75 (from HER2/neu; KIFGSLAFL, residues 369–377); ML (from calreticulin; MLLSVPLLL, residues 1–9); YM (from human papillomavirus 16 (HPV-16) E7 oncoprotein; YMLDLQPETT, residues 11–20); RL (from HER2/neu; RLLQETELV, residues 689–697); HY (from HER2/neu; HLYQGCQVV, residues 48–56); KT (from gp100 glycoprotein; KTWGQYWQV, residues 154–162); IL (from HIV-1 RT ILKEPVHGV; residues 476–484); and HA (from influenza hemagglutinin; IYSTVASSL, residues 518–526) were synthesized by the UNC Peptide Synthesis Facility (Chapel Hill, NC).

### Generation of peptide bound HLA-A2 complexes

The human MHC, HLA-A2.1, human beta-2-microglobulin (β_2_M), mouse beta-2-microglobulin, and murine H-2 K^d^ were produced as inclusion bodies in *E. coli* BL21 (DE3) (Invitrogen, Inc.). Protein was folded *in vitro* as described previously [26]. Briefly, peptide, β_2_M, and HLA-A2 heavy chain in a 10∶1∶1 molar ratio were injected into a folding buffer consisting of 100 mM Tris pH 8.0, 400 mM arginine, 2 mM EDTA, 5 mM glutathione (reduced), 0.5 mM glutathione (oxidized), and protease inhibitors PMSF, pepstatin and leupeptin. The total final protein concentration was never greater than 50 μg/ml. After incubation for 24–36 hours at 10°C the folded pMHC was concentrated in an Amicon ultrafiltration cell (Millipore, Billerica, MA) and purified using gel filtration chromatography (Phenomenex, Inc.; Torrance, CA). The purified pMHC molecules were concentrated to greater than 3 mg/ml and stored at −80°C until use. Typical yield for each 1 L refold is about 5 mg of pMHC, which is approximately a 14% yield. The pMHC was site specifically biotinylated with the biotin ligase BirA (Avidity, LLC; Aurora, Colorado) according to the manufacturer's instructions. A non-denaturing SDS PAGE gel shift assay (no reducing agent, no heat treatment) was used to confirm that the pMHCs were biotinylated.

### Construction of Fab library

We first constructed a template plasmid for constructing libraries, pFab007. This plasmid contains a modified version of the Fab-4D5 gene [27] fused to gene III of the M13 phage (corresponding to the carboxyl-terminal 208 residues of pIII). This fusion protein contains the cysteine residue of the heavy chain hinge region so as to enable bivalent display of the Fab [28]. Most residues in the CDR-L3, H1, and H2 loops were replaced with serine. A TAA stop codon and a unique BamHI site were introduced to CDR-H3. The gene was assembled from a series of synthetic oligonucleotides using PCR. The synthesized gene and the phoA promotor segment were cloned into a phage-display vector, pAS38 [29] in such a way that the heavy chain of the Fab is fused to the carboxyl-terminal 208 residue segment of M13 phage pIII. The expression of both the light chain and the heavy chain-p3 fusion were placed under the control of the phoA promotor.

**Figure 1:**
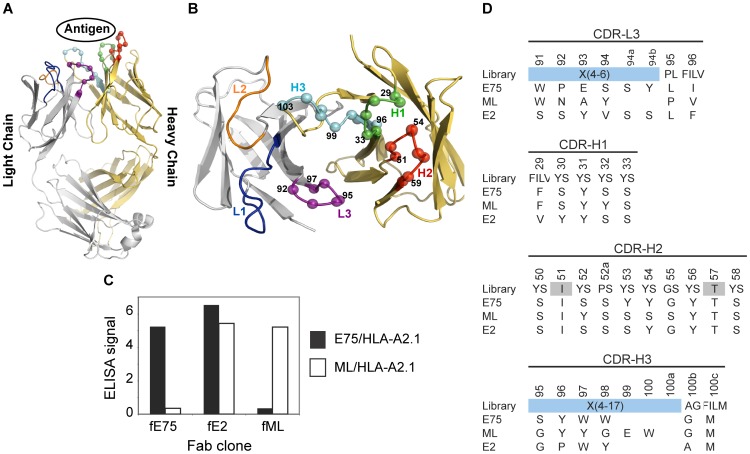
Phage-Display isolation of Fabs Specific for pMHC Molecules. Fabs specific for either E75/HLA-A2 or ML/HLA-A2 molecules were selected by phage-display technology. Each Fab construct was built from the highly stable 4D5 Fab scaffold containing both a heavy and light chain each with a single variable and constant domain (modeled from PDB ID: 1FVD) (A). The diversity of the Complementary Determining Regions (CDR) for the heavy chain; H1, H2, and H3, and the light chain; L3, was restricted in favor of tyrosine, serine, and other small amino acids. The Cα atoms of the synthetically modified H1, H2, H3, and L3 regions are shown as spheres (B). After three rounds of selection, an ELISA was used to test the specificity of the amplified clones (C). Three clones with three different specificities were identified. The Fab clones fE75 and fML bound E75/HLA-A2 and ML/HLA-A2 respectively with no detectable binding to the opposite pMHC molecule. The Fab clone fE2 showed binding to both E75/HLA-A2 and ML-HLA-A2. Following each Fab clone's specificity determination, the amino acid sequence of each clone was determined (D).

The library was constructed as follows. Oligonucleotides that encode biased amino acid mixture (indicated as “X”s in Figure 1B) for CDR-L3 and H3 were synthesized using a custom-made trimer phosphoramidite mixture (Glen Research, Sterling, VA) on an Expedite synthesizer (ABI) following instructions from Glen Research. After deprotection, the oligonucleotides were purified using acrylamide gel electrophoresis. Oligonucleotides for CDR-H1 and H2 that did not require the use of trimer phosphoramidetes were purchased from Integrated DNA Technologies. These oligonucleotides were used to introduce mutations of CDR-L3, CDR-H1, CDR-H2 and CDR-H3 using the Kunkel mutagenesis method [30]. After the initial transformation of the SS320 cells [30], the DNA for the library was purified and digested with BamHI and then used to transform the SS320 cells and produce phage particles, as described [27]. In this manner clones harboring non-mutated CDR-H3 were eliminated. This Fab library, “Library E,” contained approximately 10^10^ independent clones.

### Selection of phage-displayed antibody fragments

The library sorting was performed as previously described [27] with minor modifications. From the second round on, enriched phages were first incubated with streptavidin-coated magnetic beads, and phages that bound to the beads were removed. The “precleared” phages were then incubated with biotinylated pMHCs in solution and then captured with the streptavidin-coated magnetic beads. The pMHC concentrations used were 100, 50, 10 and 10 nM for the first, second, third and fourth rounds, respectively. Phages captured on the beads were eluted in 100 μl of 0.1 M Gly-HCl (pH 2.1) buffer and immediately neutralized with 35 μl of 1M Tris-Cl buffer (pH 8). Recovered clones were analyzed using phage ELISA and DNA sequencing as described previously [27].

### Expression and purification of soluble recombinant Fabs

The phage-display vectors for the isolated clones were converted into Fab expression vectors by inserting a gene segment encoding an 8x histidine tag and a termination codon at the 3′ end of the heavy chain gene. The carboxyl terminus of the light chain encoded a substrate tag for the biotin ligase BirA (AviTag, Avidity, LLC.). Fab proteins were expressed in the 55244 *E. coli* strain (ATCC) and purified using protein A affinity chromatography followed by cation exchange chromatography as described previously [27]. Approximately 2–5 mg of purified Fab were routinely obtained from 1 L bacterial culture. SDS-PAGE analysis showed that Fabs were in >90% purity.

### Surface Plasmon Resonance Experiments

Approximately three hundred response units (RUs) of each antibody fragment were bound to respective flow channels in a Biacore Ni-NTA sensor chip (GE Healthcare) using the 8x histidine tag on the antibody fragments. Soluble class I MHC (analyte) at concentrations ranging from 200 nM to 1 nM in two fold dilutions was injected onto the surface at a flow rate of 20 μl/min in a 60-s pulse. The NTA surface was regenerated using 0.2 M EDTA to remove all bound protein and then recharged with 0.04 M NiSO_4_. The procedure was repeated until at least three curves were obtained for each concentration of analyte. Curves obtained at each concentration were double referenced by first, subtracting the signal from the reference surface that contained no Fab from the signal for the reaction surface followed by subtraction of the average signal obtained from a set of buffer injections [31]. Data were processed using Scrubber (BioLogic Software, Campbell, Australia). The suitability of the fit was measured based on the appearance of residuals and χ^2^ values. For each Fab-pMHC binding curve, the predicted curves visually overlaid well with the experimental curves. The residuals also were small and random and χ^2^ was below 1.

### Production of Fab Tetramers

Purified Fabs were biotinylated using a site-specific biotin-ligase BirA (Avidity, LLC; Aurora, Colorado) according to the manufacturer's instructions. As described above for the biotinylation of pMHC, successful biotinylation of the Fabs was confirmed by gel shift analysis. Biotinylated Fabs were incubated with streptavidin-alexa 647 (Invitrogen, Inc.) at a 4 to 1 molar ratio for ten minutes at room temperature to make the Fab tetramers.

### Flow cytometric analysis of peptide-loaded HLA-A2 on cell surface

T2 cells are deficient in the TAP1 and TAP2 proteins that are responsible for transporting antigenic peptides from the cytoplasm to the endoplasmic reticulum, but are able to be loaded with exogenous peptides for loading of HLA-A2 molecules on their cell surface. T2 cells (1×10^6^/ml) were incubated in RPMI medium and incubated with one of the peptides E75, ML, IL, YM, RL, HL, KT, or IL (50 µM) overnight. After the incubation, the cells were washed to remove the excess peptide and incubated with either BB7.2 Ab (0.5 mg/ml) to detect the level of HLA-A2 molecules present on the surface or the complex of biotinylated fE75 or fML1 with streptavidin-alexa 647 (Invitrogen, Inc.) for 30 min. at 4°C. Incubating the T2 cells with peptides leads to an observable increase in BB7.2 staining compared with untreated cells. In all experiments, the IgG2b isotype control was included for determining nonspecific binding. Flow cytometry data were analyzed using Summit software (Beckman Coulter, Inc.).

### Tumor Cell Staining

All adherent tumor cell lines were detached from the tissue culture flask using 1× trypsin/EDTA (Sigma-Aldrich, LLC). Cells were washed and then incubated with anti-HER2/neu antibody (Anti-c-erb B-2 Ab-2, 9G6.10) for 30 min. at 4°C followed by incubation with Alexa Fluor 647 labeled Goat anti-mouse IgG1 and PE labeled HLA-A2 clone BB7.2 to analyze the phenotype of each tumor cell line. Separately, each tumor cell line was prepared as before, but stained with Fab-Alexa Fluor 647-streptavidin tetramer for 30 min. at 4°C. After the final staining incubation, cells were washed and analyzed by flow cytometry (Cyan, Beckman Coulter, Inc.). Pearson's correlation coefficient and multiple linear regression analysis were completed for the mean and median fluorescence intensity observed for each cell line's HLA-A2, Her2/neu, and Fab staining. No difference was observed whether mean or median fluorescence was used for the analysis.

### DOTA Conjugation and ^64^Cu Radiolabeling

All solvents were prepared from 18MΩ water and eluted from a column of chelex 100 (Serological Research Institute) to remove metals from all buffers. Free metals were removed from purified Fabs by dialysis twice with 1L of 0.2 M NaHCO_3_ at pH 8.2. Fabs were conjugated to S-2-(4-Isothiocyanatobenzyl-)1,4,7,10-tetraazacyclo-dodecane-tetraacetic acid (DOTA-NCS) purchased from Macrocyclics (Dalax, TX) by using the isothiocyanate linkage method as described previously [32]. The molar ratio of the DOTA conjugate to Fab used was 5∶1. The reaction proceeded at pH 8.2 in 0.2 M NaHCO_3_ over 18 hours at room temperature. Unconjugated DOTA was removed from the reaction by a PD-10 desalting column (GE Healthcare). The average number of chelates per Fab was determined as described previously [33]. The positron emitting isotope ^64^Cu (copper chloride in 0.1 mol/L HCl; radionuclide purity, >99%) was provided by Mallinckrodt Institute of Radiology (Washington University School of Medicine, St. Louis, WA). The DOTA-conjugated Fab (20 μM) was incubated with approximately 1.5 mCi of ^64^CuCl_2_ in 1.0 mM citric acid (pH 5.5). The solution was incubated for 1 hr at 40°C. The reaction was monitored by TLC (silica) and developed with 0.1 M ammonium acetate (25%)/0.001 M citric acid (25%)/methanol (50%). Free ^64^Cu was complexed as ^64^Cu citrate with an Rf >0.5 and ^64^Cu DOTA-Fab had an Rf <0.5. The radiochemical yield was determined to be >97.0%.

### Cell Binding Assay of Radiolabeled fE75 Fab

The SKOV3 cells transfected with HLA-A2 were harvested as previously described for tumor cell staining. The cells were incubated with the purified ^64^Cu DOTA-Fab at increasing concentrations for 30 min. at 4°C. As a demonstration of specificity, the SKOV3 transfected HLA-A2 cell line was incubated with ^64^Cu DOTA-Fab at increasing concentrations and 300 nM soluble E75/HLA-A2 pMHC. Following the incubation, the cells were washed twice and then the bound radiolabeled fE75 counts per minute (cpm) was measured with a 2470 WIZARD^2^ automatic gamma counter (PerkinElmer Inc.).

### Micro Positron Emission Tomography/Computed Tomography (microPET-CT) Imaging in Tumor-bearing mice

The preliminary experiments used NOD.SCID (SCID) mice with single tumors injected subcutaneously into the flank of the mouse. SCID and HLA-A2 transgenic NOD.SCID mice were a gift from Dr. Jeffrey Frelinger (Department of Immunobiology, University of Arizona) [34]. All SCID mice were injected subcutaneously on either the right or left flank with either negative control tumors 1×10^6^ SKOV3 (4 mice) or positive tumors SKOV3 transfected HLA-A2 cells (3 mice). Following these experiments, we used HLA-A2 transgenic SCID mice and because of limitations in the number of available mice, injected both tumors on opposite flanks of each mouse. The HLA-A2 transgenic SCID mice were injected with 1×10^6^ MDA-MB-231 cells and SKOV3 cells on opposite flanks (3 mice). Mice were monitored every other day by palpation and the size of tumor growth was measured. Once the tumor growth could be observed by palpation, mice were injected with the Fab and imaged using a microPET-CT (eXplore Vista PET-CT, GE HealthCare, Inc.). Mice were injected with approximately 20 μg of ^64^Cu-DOTA-Fab (specific activity: 50–60 μCi/μg) in PBS via the tail vein. For all scans, mice were anesthetized using 2% isoflurane, positioned in a prone position along the long axis of the microPET scanner and imaged. Dynamic PET acquisitions were taken over the first hour for selected mice. Static acquisitions were taken for 10 min. at one-hour post injection for all mice. All mice were additionally imaged by micro computed tomography (microCT) prior to the microPET scan in the same bed position for anatomical reference. Following the imaging, tumors were excised, cryopreserved with liquid nitrogen, and cryosectioned for radiographic analysis. PET images were reconstructed using an ordered subsets-expectation maximization (OSEM) algorithm with scatter, random, and attenuation corrections, and the PET pixel size was 0.3875×0.3875×0.775 mm [35]. Standardized uptake values (SUVs) were calculated pixel-wise by normalizing for injected dose and animal mass. The PET images were coregistered with the microCT images using the scanner software for identification of anatomical structures. Reconstructed microPET and microCT images were viewed and regions of interest (ROIs) drawn and SUVs quantified using AMIDE [36]. Tumor ROIs were drawn manually to avoid adjacent organs and tumor metastases because the metastases typically had very poorly defined borders. Average SUV was measured for each specified ROI. The student's t test was used to compare the SUVs from single tumor bearing SCID mice. The paired student's t test was used to compare the SUVs from the tumors of the double tumor-bearing, HLA-A2-transgenic SCID mice.

## Results

### Generation of synthetic antibody-fragments specific to pMHC complexes

Our goal is to develop tools to target specific tumor cells *in vivo* for diagnostic testing and treatment. We hypothesized that peptides bound to MHC would be good targets for such tools. The HER2/neu peptide E75 (KIFGSLAFL) was selected for analysis based on its clinical relevance to HER2/neu over-expression [37]–[39] and the success of E75 peptide vaccines and targeting using E75 in peptide-pulsed dendritic cell immunization therapies [40], [41]. It has been notoriously difficult to create monoclonal antibodies (mAb) to specific peptide/MHC complexes, presumably because of the sequence conservation between MHC molecules injected and present in the immunized mouse [42].

As an alternative to conventional mAb production, we sought to isolate antibody-fragments (Fabs) specific for peptide-bound MHC molecules using phage-display technology. We utilized a “synthetic” antibody library built on a highly stable 4D5 Fab scaffold (Figure 1A) [43] in which the amino acid diversity in the Complementarity Determining Regions (CDR) was designed using simple but highly tailored amino acid mixtures [27]. Because such synthetic antibody libraries are not subjected to clonal selection against self-antigens, unlike common recombinant antibodies derived from natural immune systems, we felt that synthetic antibody libraries, coupled with phage-display selection, offered a particularly effective approach to generating antibodies to pMHCs. Based on our previous work [27], we constructed a new antibody library in which four of the six CDRs were diversified, as described in Figure 1B. This library is similar to “Library D” of Fellouse et al. [27], but its amino acid diversity in CDR-L3 was expanded and also the composition of the expanded amino acid diversity, designated as X in Figure 1, was modified based on the analysis of antibody clones isolated from Library D [27].

We used the HER2/neu derived peptide, E75 (KIFGSLAFL), or calreticulin derived peptide, ML (MLSVPLLL), bound to the human MHC molecule HLA-A2 as the targets for sorting the synthetic antibody library. After four rounds of selection, an ELISA was used to test for specificity of the amplified phage Figure 1C. We identified three distinct classes of antibodies: (i) selective to E75/HLA-A2, (ii) selective to ML/HLA-A2, and (iii) cross-reactive to the two pMHCs. Two clones, fE75 and fML, selective to E75/HLA-A2 and ML/HLA-A2 respectively, were chosen for further study based on the phage ELISA, which identified each phage clone to have high affinity and specificity for their respective pMHC. As expected, the two clones have distinct CDR sequences (Figure 1D).

**Figure 2:**
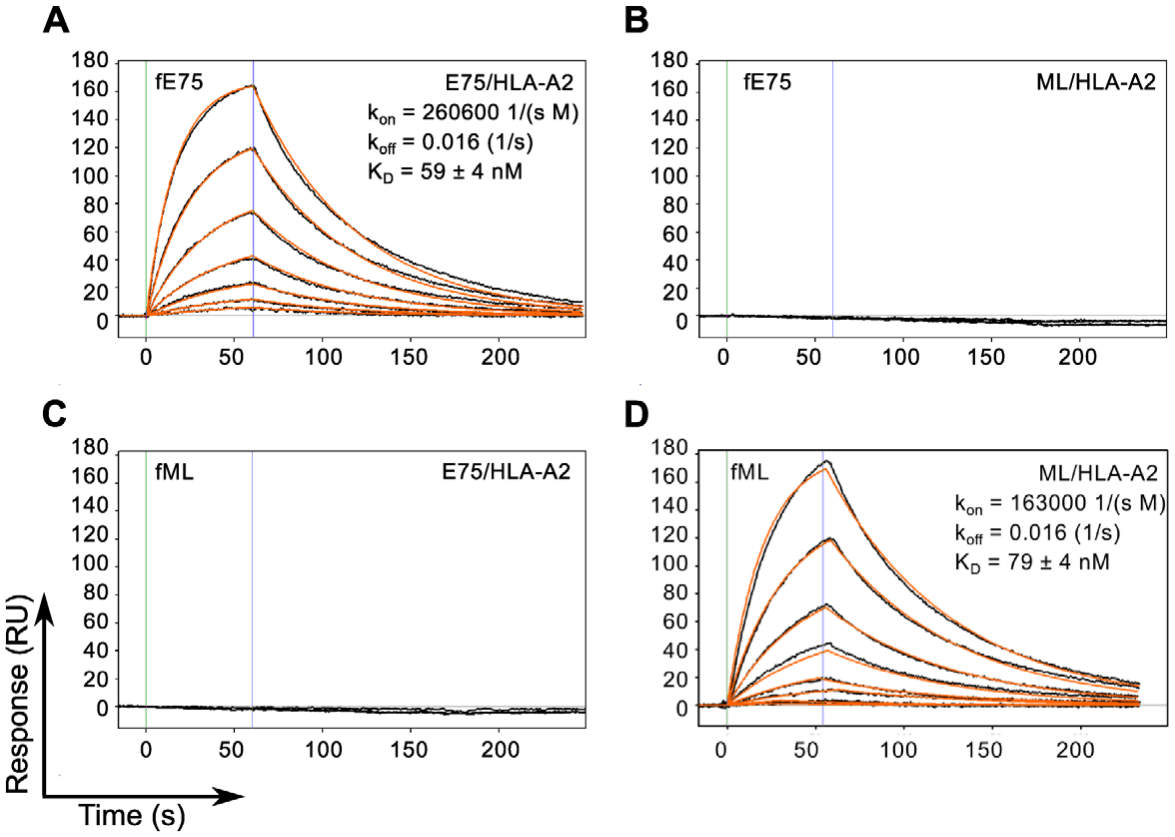
TCR-like Fabs Bind Cognate pMHC with Nanomolar Affinity. SPR binding response curves of fE75 binding E75/HLA-A2 (A) and ML/HLA-A2 (B) and fML binding E75/HLA-A2 (C) and ML/HLA-A2 (D) are shown. Each Fab was immobilized onto individual flow channels in an NTA-Ni chip. Kinetic data for each Fab binding each pMHC molecule were globally fit to a bimolecular reaction. Green and blue lines designate the start and end respectively of each pMHC injection. Binding curves and curve fits are drawn in black and orange respectively. Each binding curve represents a different concentration of pMHC beginning at 200 nM and decreasing to 1.5 nM in 2 fold dilutions. No binding was observed for either Fab binding non-cognate pMHC molecules up to 400 nM.

To assess the binding affinity, kinetics and specificity of the two Fabs, they were produced as soluble proteins, with a His-tag at the C-terminus of the heavy chain and a biotinylation tag at the C-terminus of the light chain and characterized using Surface Plasmon Resonance. The results shown in Figure 2 demonstrate that the model appropriately describes the binding responses of each Fab for its cognate pMHC molecule, with curve fitting residuals at or below 1 RU. The K_D_ value for fE75 binding E75/HLA-A2 was determined to be 59 +/− 4 nM based on three separate experiments (Figure 2A). The K_D_ for fML binding ML/HLA-A2 was 79 +/− 4 nM based on three separate experiments (Figure 2D). No binding was observed for fE75 and fML binding the non-cognate pMHC (Figure 2B, 2C), or to other non-cognate pMHC molecules that differed in peptide alone or unrelated peptide bound to unrelated MHC (data not shown).

### Fabs bind to cell surface pMHC

**Figure 3:**
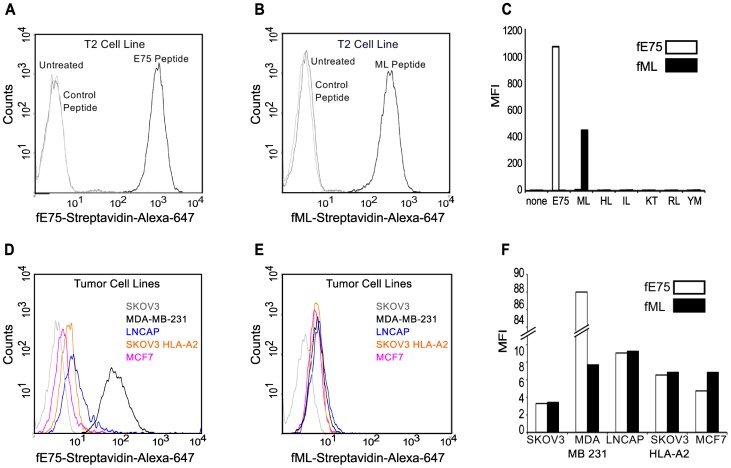
Fabs bind specifically to endogenously processed and presented levels of pMHC molecules. Flow cytometric analysis revealed that the Fab tetramer, composed of biotinylated fE75 (A) or fML (B) and streptavidin-Alexa-647, bound to T2 cells incubated with the cognate peptide, but not to control peptides or untreated T2 cells in three separate experiments. In C, a graph of the MFI for several other control peptides against the cognate peptide for each Fab was shown. The staining of HER2/neu positive tumor cell lines by the fE75 and fML tetramers was shown in D and E respectively. The graph of the MFI for one representative experiment from four total was shown in F.

In order to test whether these Fabs can be used to find specific pMHC on the cell surface, Fab-displaying streptavidin tetramers were made and used to bind to peptide pulsed T2 cells. The T2 cell line is deficient in pMHC cell surface expression due to the absence of the transporter associated with antigen processing (TAP1 and TAP2). The MHC HLA-A2 on the surface of T2 cells is more “peptide-receptive” than on normal cells and therefore; specific peptides may be loaded onto HLA-A2 by incubating these cells with exogenous peptides [44]. T2 cells were incubated with ML, E75 or control peptides. The biotinylated Fabs were respectively conjugated to fluorescently-labeled streptavidin to form “Fab tetramers”. The fE75 tetramer bound to T2 cells incubated with E75 (Figure 3A), but not to untreated cells. Similarly, the fML tetramer bound to T2 cells incubated with ML (Figure 3B), but not to untreated cells. Additionally, neither Fab bound to T2 cells incubated with any of the control peptides (Figure 3C). Therefore, in agreement with the SPR experiments, these Fabs bound specifically to their cognate pMHC expressed on the cell surface.

### The Fabs detect endogenously processed and presented pMHC complexes

Addition of exogenous peptide to T2 cells generates a much higher density of the particular peptide/MHC molecule on the cell surface compared with endogenous levels found on normal TAP expressing cell types [45], [46]. Thus, we evaluated the ability of fE75 and fML to recognize physiological levels of peptide presented by the HLA-A2 molecule using flow cytometric analysis of several human tumor cell lines: MDA-MB-231 breast tumor cells (HLA-A2^pos^ and HER2/neu^pos^); SKOV3 HLA-A2 transfected ovarian tumor cells (HLA-A2^pos^ and HER2/neu^pos^); MCF7 breast tumor cells (HLA-A2^pos^ and HER2/neu^pos^); LNCaP prostate tumor cells (HLA-A2^pos^ and HER2/neu^pos^); and negative control SKOV3 ovarian tumor cells (HLA-A2^neg^ and HER2/neu^pos^). All the cell lines express HER2/neu at varying levels. Thus, as anticipated, the HLA-A2 positive human cell lines showed variable fE75 binding compared to the negative control tumor cell line SKOV3 (HLA-A2^neg^) as shown in Figure 3D. All of the HLA-A2 positive cell lines had similar binding of the fML Fab (Figure 3E), but comparable to fE75, neither Fab bound to the HLA-A2 negative control cell line SKOV3 (Figure 3F). In addition, a further control was added with a CHO cell line transfected with HLA-A2. This cell line was HLA-A2 positive but HER2/neu negative. No binding of either fE75 or fML1 was observed for this cell line (data not shown).

**Figure 4:**
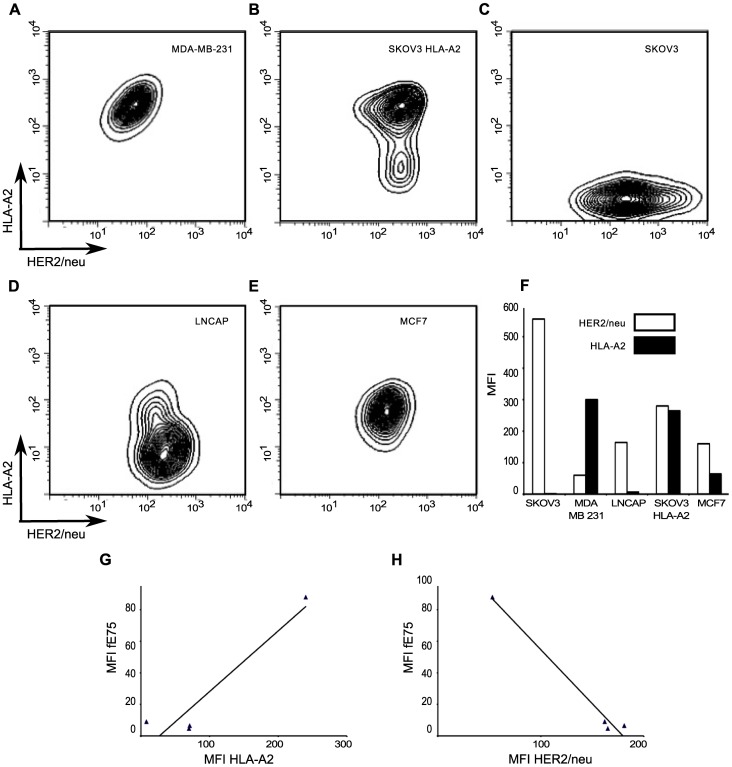
HLA-A2 and HER2/neu expression is high variable on each tumor cell line. The surface expression of HLA-A2 and HER2/neu for each human tumor cell line was measured by flow cytometry. The representative plots of HLA-A2 versus HER2/neu for each tumor cell line were shown in A-E. In F, a graph of the MFI of one representative experiment from four total was shown. G and H were plots of the MFI of fE75 versus the MFI of HLA-A2 or MFI of HER2/neu respectively. Simple two-dimensional linear regression analysis models do not model the system well. The black lines showed weak correlation of fE75 MFI with HLA-A2 (r^2^ = 0.40) or HER2/neu expression (r^2^ = 0.62).

Next, we sought to understand the cause for the differences in fE75 binding to the tumor cell lines in order to improve our ability to use the TCR-like Fabs as diagnostic and treatment tools. The cell surface level of peptide/HLA-A2 complexes is dependent on multiple parameters including: protein antigen availability, peptide generation by the proteasome, antigen processing, and MHC expression [47]. The data allow us to determine which protein plays a more important role in the level of any particular pMHC complex at the cell surface: MHC (HLA-A2) or antigen (HER2/neu). We measured HER2/neu and HLA-A2 expression by flow cytometry. There were significant differences in both HLA-A2 expression and HER2/neu levels between the cell lines (Figure 4A–F). The MDA-MB-231 cell line had the highest level of HLA-A2 expression, but also the lowest HER2/neu levels. Both MCF7 and LNCaP cell lines had intermediate levels of HER2/neu, but lower expression of HLA-A2. The SKOV3 HLA-A2 cell line had the highest level of HER2/neu and the second highest MFI of HLA-A2.

Simple two-dimensional linear regression analysis models showed weak correlation of fE75 MFI with HLA-A2 (r^2^ = 0.40) and weak, but stronger correlation with HER2/neu expression (r^2^ = 0.62) (Figure 4 G-H). Both of the regression analyses were dominated by the contribution made by MDA-MB-231. If that cell line were removed from the analysis, no correlation exists for fE75 MFI and HLA-A2 (r^2^ = 0.09) nor HER2/neu (r^2^ = 0.00). Since that is not meaningful biologically and we know that fE75 binding did vary for each cell line, we know that either there was another unknown important factor or that both HLA-A2 and HER2/neu influenced fE75 binding. The coordinated involvement of both HLA-A2 and HER2/neu on fE75 levels was modeled using multi-parameter linear regression analysis [48]. This model gave an excellent fit for the correlation of fE75 binding using both HLA-A2 and HER2/neu expression levels (r^2^ = 0.97). Removing the MDA-MB-231 cell line from this analysis showed that there was still a significant correlation (r^2^ = 0.89), suggesting that even though MDA-MB-231 appears to dominate the regression, the correlation between fE75 binding and both HLA-A2 and HER2/neu is valid even without the MDA-MB-231 cell line. These data show that both MHC expression levels and the antigen expression affect pMHC expression on tumor cell lines. Both MHC and source of the peptide antigen must be considered in order to accurately predict pMHC expression levels.

### The TCR-like Fab recognizes E75/HLA-A2 positive tumors in a xenotransplanted-tumor mouse model

To address whether the TCR-like Fabs can be used *in vivo*, we used positron emission tomography-computed tomography (PET-CT), which provides excellent sensitivity to radiolabels, allowing quantification of the uptake of the imaging agent at the tumor [49]. The Fab, fE75, was made into a PET imaging agent by attaching a chelator, S-2-(4-Isothiocyanatobenzyl)-1,4,7,10-tetraazacyclo-dodecane-tetraacetic acid (DOTA-NCS). The DOTA chelator provides flexibility as it can chelate both ^64^Cu for PET and ^111^In for single-photon emission computed tomography (SPECT). We routinely obtained an average of 0.7 moles of DOTA per mole of fE75. The yield of ^64^Cu- fE75-DOTA conjugate was obtained in greater than 95% purity as determined by thin layer chromatography. To confirm that addition of this chelator and the radioactive metal did not alter the binding affinity of fE75 for E75/HLA-A2, in *vitro* cell binding studies were performed. The ovarian tumor cell line SKOV3 transfected with HLA-A2 was incubated with a range of concentrations of radiolabeled Fab. A Scatchard analysis was used to calculate a binding affinity of 111 nM (95% CI: 63–159), which was in good agreement with the SPR data (59 +/− 4 nM). The ^64^Cu- DOTA-fE75- conjugate maintained its specificity for E75/HLA-A2, as soluble recombinant E75/HLA-A2 complexes were able to block binding of the radio-labeled fE75 to SKOV3/HLA-A2 cells (Figure S1).

**Figure 5:**
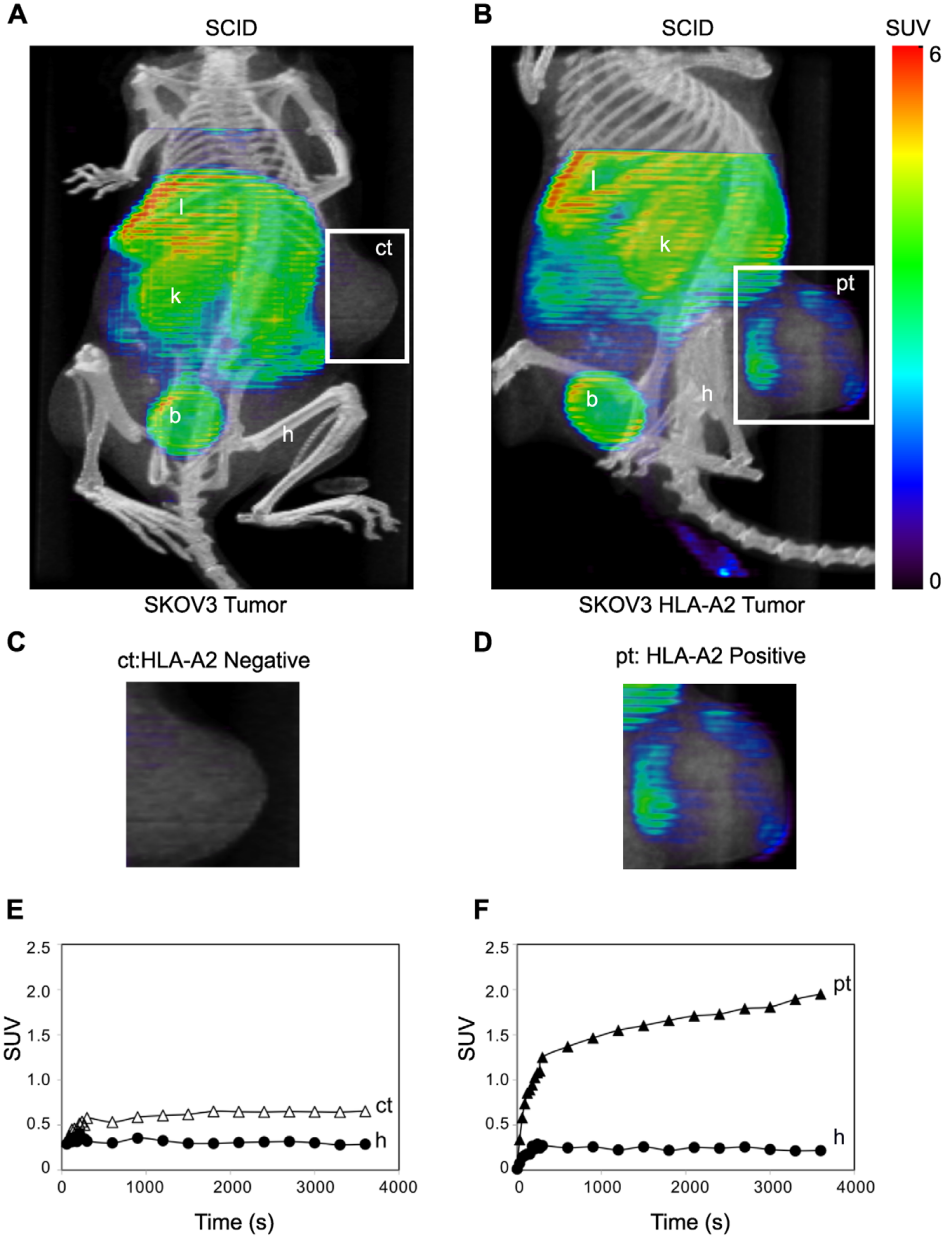
TCR-like Fab bind specifically to human tumor cells in SCID mice. Mice were injected intravenously with ^64^Cu-DOTA-fE75, which binds to tumors expressing both HLA-A2 and HER2/neu *in vivo*. Coronal views of small-animal PET images with coregistered CT images of SCID mice bearing human tumor (SKOV3 or SKOV3 HLA-A2) xenografts (A and B) in flanks at 1 h post-injection. Intensities of PET slices were scaled to the same maximum. Cropped views of only the tumors designated by the white boxes for the mice presented in A and B were shown in C and D respectively. The standardized uptake values (SUV) for ^64^Cu-DOTA-fE75 in the tumors and selected organs over time were shown below each respective mouse (E and F). B  =  bladder, h  =  humeral muscle, k  =  kidneys, l  =  liver, ct  =  control HLA-A2 negative tumor; SKOV3 cell line, pt  =  HLA-A2 positive tumor; SKOV3 HLA-A2.

The first test of uptake of the labeled fE75 used SCID mice. These mice do not express either human HER2/neu or HLA-A2. The question asked is whether once the Fab is injected into the tail vein, will it be able to accumulate in the tumor. The ^64^Cu- DOTA-fE75 conjugate was injected via the tail vein of severe combined immunodeficiency (SCID) mice bearing either xenotransplanted SKOV3 or SKOV3 HLA-A2 tumors. MicroPET (color) and microCT (black and white) images of the mice were collected and overlayed (Figure 5A and5B). The xenografted human tumors on either the left or right flanks were clearly identifiable in all microPET/CT images. The relatively small size of the Fab (∼55 kDa) was below the renal clearance threshold of approximately 64 kDa meaning that it should be rapidly eliminated by renal clearance [50]–[55]. Literature indicated that the large signal from the liver might be attributed to Cu/Zn superoxide dismutase (SOD1), chelation of the ^64^Cu [56], [57]. Tumors have been shown to trap injected materials non-specifically [58], but, there was very little uptake of ^64^Cu-DOTA-fE75 in the HLA-A2 negative SKOV3 control tumor (Figure 5A, C; labeled ct) suggesting that this is not a significant problem in our model. In contrast, there was easily observable uptake of the fE75 imaging agent in the HLA-A2 positive tumor (Figure 5B, D; labeled pt). Even after one hour, increased up-take at the HLA-A2 positive tumor sites was observed, but not at the negative control tumor. Accumulation of ^64^Cu-DOTA-fE75 was observed at the edges of the tumor that suggested at first that the Fab did not penetrate the tumor well. However, visual inspection of the sectioned tumor showed necrosis in the center of the tumor and radiography of the tumors (Figure S2) showed that the Fab was bound appropriately on the outside where live tissue remains. PET/CT images of additional human tumor bearing SCID mice were shown in Figure S3. These data showed that the Fab does accumulate at the site of the correct tumor.

Dynamic scans over the course of sixty minutes (Figure 5E and 5F), show that there is an initial rapid accumulation of ^64^Cu- DOTA-fE75 followed by a steady-state in all the tissues (liver, kidney, humoral muscle, and control tumor) except for the HLA-A2 positive tumor. The HLA-A2 positive tumors showed initial rapid accumulation followed by a steady rise in the ^64^Cu- fE75-DOTA signal suggesting that specific binding occurs. The ratio of the slope of the Standard Uptake Values (SUVs) from 300 to 3600 sec. for the HLA-A2 positive tumors compared to the HLA-A2 negative control tumors was 6.6. This was expected if ^64^Cu-DOTA-fE75 were to accumulate due to binding to its cognate pMHC, E75/HLA-A2, at the HLA-A2 positive tumors. Furthermore, an unpaired t test analysis of the SUVs of the ^64^Cu-DOTA-fE75 signal sixty minutes post injection gave a statistically significant difference between the HLA-A2 positive tumors and the HLA-A2 negative tumors (p<0.004, n = 4). Together these experiments demonstrate that the fE75 Fab can accumulate in tumors that present E75/HLA-A2 on their cell surface *in vivo*.

**Figure 6:**
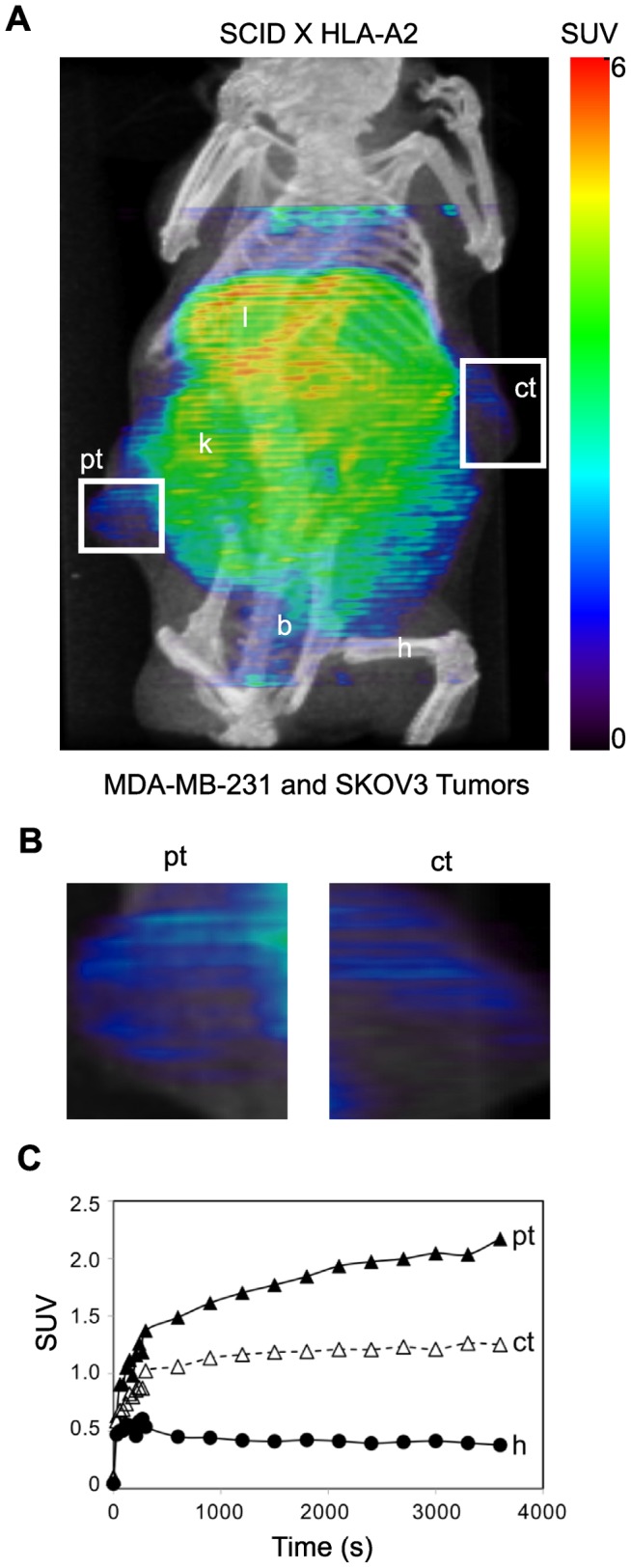
TCR-like Fab bind specifically to human tumor cells in HLA-A2 transgenic SCID mice. Similarly as describe for the SCID mice experiments (Figure 5), HLA-A2 transgenic SCID mice were injected intravenously with ^64^Cu-DOTA-fE75. Coronal views of the small-animal PET image with coregistered CT images of a scid HLA-A2 transgenic mouse bearing human tumor (MDA-MB-231 and SKOV3) xenographs (A) in flanks at 1 h post-injection. Intensities of PET slices were scaled to the same maximum. Cropped views of only the tumors designated by the white boxes were shown in B respectively. The standardized uptake values (SUV) for ^64^Cu-DOTA-fE75 in the tumors and selected organs over time were shown in C. B  =  bladder, h  =  humeral muscle, k  =  kidneys, l  =  liver, ct  =  control HLA-A2 negative tumor; SKOV3 cell line, pt  =  HLA-A2 positive tumor; MDA-MB-231 cell line.

While the experiments above show that ^64^Cu-DOTA-fE75 can target a pMHC *in vivo*, they do not mimic an important consideration for human use. In humans, most cells in the body express HLA-A2 bound numerous different peptides found in each cell type [59], [60]. If the ^64^Cu-DOTA-fE75 imaging agent has a small degree of non-specific binding to HLA-A2 with other peptides, we would not see accumulation at the tumor because the Fab would be greatly diluted by the nonspecific binding before arriving at the tumor. Thus, we used an HLA-A2 transgenic mouse crossed to the SCID background, which allows for the tumors to grow and for all of the mouse cells to express HLA-A2. The HLA-A2 transgenic SCID mice were xenografted with the SKOV3 (control tumor, ct) and MDA-MB-231 (positive tumor, pt) (Figure 6A, B and Figure S3C). Dynamic scans were obtained over sixty minutes following tail-vein injection of ^64^Cu-DOTA-fE75 (Figure 6C). The dynamic curves from the HLA-A2 transgenic SCID mice were similar to what was seen in the SCID mice that did not express HLA-A2 (Figure 5E–F). The ratio of the calculated slopes of the SUVs from 300–3600s for the HLA-A2 positive tumor compared to the HLA-A2 negative control tumor was 3.8, again indicating positive accumulation of ^64^Cu-DOTA-fE75 at the HLA-A2 positive tumor. Furthermore, comparison of the SUVs sixty minutes post injection showed that the human tumors positive for HLA-A2 and HER2/neu had statistically significant uptake and retention of ^64^Cu-DOTA-fE75 compared to the negative control tumor SKOV3 (paired t test, p<0.03, n = 4). This is a significant achievement in specificity because the ^64^Cu- fE75-DOTA was exposed to millions of HLA-A2 molecules with various peptides and still accumulated at the HLA-A2 positive tumors.

## Discussion

Our long-term goal is to construct a generalized approach to specifically target tumors *in vivo* for diagnostic and therapeutic use. We have used imaging as our first goal because we rationalize that if we can image specifically, we can target therapeutics specifically. We began with the observation that the immune system is fully competent to target tumors *in vivo* [61]–[70]. A major portion of this response is composed of cytotoxic T cells. T cells are able to recognize peptides derived from proteins in the cells, bound to MHC molecules (pMHC) using their clonotypic T cell receptor [71], [72]. Unfortunately for the patient, the tumor infiltrating T cells do not always kill the tumors. As a substitute for tumor specific recombinant T cell receptors, monoclonal antibodies that recognize specific pMHC complexes have been generated [24], [45], [46], [73]–[80], but usually have low affinities compared to those isolated from bacteriophage libraries [78], [81]–[83]. Rational design of high affinity antibodies directed towards pMHC has yielded affinities in the nanomolar range [84], but that approach is laborious and must be done for each antibody individually.

The Fabs isolated from our synthetic antibody-fragment library have binding affinities in the mid-nM range, consistent with a previous work demonstrating the ability of these synthetic libraries to generate high-affinity antibodies without the need for affinity maturation [27]. The level of affinity of our Fabs is approximately 1000 times better than that for TCRs binding pMHC, which is typically in the μM range [85]. This is also roughly a 100-fold improvement over TCR-like monoclonal antibodies produced from classical hybridoma technology [86]. These Fab have affinities similar to other peptide/HLA-A2-specific antibodies isolated from bacteriophage libraries [87], [88].

Previous studies have suggested that the higher affinity of TCR-like Fab compared to T cell receptor is due to faster on-rates instead of slower off-rates [89]. Our data are partially consistent with this concept as our Fabs bind cognate pMHC with faster rates of association, but our Fabs also show significantly slower rates of dissociation. Therefore, increased affinity of TCR-like Fabs is not solely due to increased on-rates as has been suggested.

Using these Fabs, we have shown that predicting pMHC levels on the cell surface is dependent highly on both MHC and HER2/neu antigen expression. Different tumor cell lines showed varying levels of fE75 binding/staining because they have highly different levels of HLA-A2 and HER2/neu. In agreement with previous data [90], we observed no direct correlation between levels of E75/HLA-A2 detected by fE75 and HER2/neu detected by mAb suggesting that HER2/neu levels are not directly correlated with E75/HLA-A2 levels on the cell's surface. However, multiple components are involved in the levels of any particular pMHC and often these components may affect one another. Logically, it seems likely both HER2/neu and HLA-A2 levels would affect E75/HLA-A2 levels on the cell surface. Multi-parameter linear regression analysis yielded a strong correlation between fE75 binding and the both HLA-A2 and HER2/neu expression. We conclude that both variables, the source protein and MHC levels, are both highly important.

The fE75 Fab was demonstrated to bind selectively to HLA-A2 positive tumors *in vivo* in SCID mice. Thus, fE75, a TCR-like Fab, can be used *in vivo* and potentially used as a drug delivery vehicle. This is consistent with other reports that demonstrate that tumors xenografted in SCID mice were reduced or even eliminated as via complement fixation or induction of apoptosis [24], [91]. Moreover, a TCR-like Fab fused to a truncated form of *Pseudomonas* exotoxin, PE38KDEL showed *in vivo* dose dependent xenografted tumor killing in SCID mice [87], [92]. Immunotherapy and drug deliver by TCR-like Fabs is possible only if the specificity of the Fab is exceptional and does not bind to similar pMHC presented on healthy cell surfaces.

However, our tests went one important step farther than those performed previously. We showed that these TCR-like Fabs can function similarly to human T cells; they can be highly selective *in vivo* even in the context of human HLA-A2 expressed in HLA-A2. Even with the thousands of peptide/HLA-A2 complexes available on healthy cells of the HLA-A2 transgenic SCID mouse, the Fab still accumulated at the transplanted tumors. This is especially interesting considering that the sequence of the peptide E75 from HER2/neu is identical to that found in mouse epidermal growth factor receptor 2 [93] and that the HLA-A2 expression in the HLA-A2 transgenic mouse model is expressed at comparable levels to H-2D^b^ in thymus, bone marrow, and spleen [94]. The dynamic scans showed initial rapid accumulation of ^64^Cu- DOTA-fE75 followed by a steady-state in all the tissues (liver, kidney, humoral muscle, and control tumor) except for the HLA-A2 positive tumor similar to the SCID mouse experiments. It is not clear why we did not see accumulation of the Fab in sites other than the tumor and those predicted by the injection of this sized protein. Although the mouse antigen presenting machinery can process and present peptides recognized by human HLA-A2-restricted T cells [93], it is possible that there are differences that preclude presentation of the E75 peptide or that the expression level is so much lower in normal cells that there is a very small dilution of the signal compared to the tumor.

Theoretically, the best *in vivo* images of the ^64^Cu-DOTA-Fab accumulation would occur after removal of the unincorporated label, but the half-life of fE75 binding to E75/HLA-A2 measured by SPR is on the order of minutes and due to the high vascularization of the tumors, the Fab is removed readily. We conclude that all of these Fab sized probes of roughly 55 kDa could be highly specific and bind very tightly, but they do not have a long enough half-life to be useful on their own unless they are endocytosed by the cell. The signal could be greatly increased by multimerization of the Fab [95], [96]. However, this problem does not diminish the significance of the observation that the Fab targeting was successful even in the context of transgenic expression of HLA-A2 in all the cells of the mouse.

Antibodies specific for pMHC are useful tools for understanding antigen presentation *in vivo* and allowing targeted therapy for cancer. Not only can they be used to directly visualize T-cell epitopes [45], [90], [97], they can aid in method development for peptide-based immunotherapy, where visualization of a particular pMHC before and after treatment would be beneficial. This can include analysis of intracellular generation, trafficking, cell surface expression, and stability of pMHC complexes on antigen presenting cells (APC). Antibody fragments with pMHC specificity can improve on the successes of adoptive T-cell therapy. Even though up to 50% of patients treated with *ex vivo* expanded tumor-infiltrating lymphocytes show clinical responses [70], [98], it is not possible to produce significant quantities of tumor infiltrating T cells from all cancer patients due to the inability to either isolate or expand them *in vitro*. There is promise in the field of genetically modified T-lymphocytes, where TCR-like antibodies are transferred to T lymphocytes via retroviral infection for their expression on the T cell surface [99]. T cells expressing tumor-specific receptors, chimeric antigen receptors and modified TCRs specifically killed tumor cells and had antitumor effects *in vivo* [100]–[103].

In summary, we have demonstrated that pMHC specific Fabs can be quickly and efficiently isolated from our skewed bacteriophage library. These particular Fabs provide much greater stability and higher affinity than would T cell receptors making them particularly good imaging agents. One of the Fabs, fE75, shown to have high affinity and high specificity, was derivitized as an *in vivo* imaging agent and selectively localized to HLA-A2 positive tumors in SCID HLA-A2 transgenic mice. It is anticipated that phage display-derived TCR-like antibody fragments have a wide range of applications in research, diagnostic, and therapeutic applications.

## Supporting Information

Figure S1 **Saturation** (**•**) **binding curves of ^64^Cu-DOTA-fE75 and SKOV3 HLA-A2** (**E75/HLA-A2 pMHC positive**) **cells.** Each data point represents the mean ± standard error of the mean of triplicate measurements. The K_D_ of ^64^Cu-DOTA-fE75 was determined to be 111 nM (95% CIs: 63–159). ^64^Cu-DOTA-fE75 binding (Ο) to SKOV3 HLA-A2 cells blocked by 300 nM soluble E75/HLA-A2 pMHC.
(TIF)

Figure S2 **Radiography images of excised human tumors from ^64^Cu-DOTA-fE75 injected SCID and HLA-A2 trangenic SCID mice.** HLA-A2 positive and HLA-A2 negative tumors from SCID mice were shown in A and B. HLA-A2 positive and HLA-A2 negative tumors from HLA-A2 transgenic SCID mice were shown in C–F.
(TIF)

Figure S3 **Additional PET/CT images of SCID and HLA-A2 transgenic SCID mice.** Mice were injected intravenously with ^64^Cu-DOTA-fE75, which binds to tumors expressing both HLA-A2 and HER2/neu *in vivo*. Coronal views of small-animal PET images with coregistered CT images of SCID mice bearing human tumor (SKOV3 or SKOV3 HLA-A2) xenografts (A and B) or HLA-A2 transgenic SCID mouse bearing two tumors (SKOV3 and MDA-MB-231) xenografts (C) in flanks at 1 h post-injection were shown. Intensities of PET slices were scaled to the same maximum. Cropped views of only the tumors designated by the white boxes for the mice presented in A, B, and C were shown in D, E and F respectively. B  =  bladder, h  =  humeral muscle, k  =  kidneys, l  =  liver, ct  =  control HLA-A2 negative tumor; SKOV3 cell line, pt  =  HLA-A2 positive tumor; SKOV3 HLA-A2 or MDA-MB-231.
(TIF)
