# Supplementary figures and images for: T Cell Receptor-Like Recognition of Tumor In Vivo by Synthetic Antibody Fragment
Source: PLoS One. 2012 Aug 20;7(8):e43746. doi: 10.1371/journal.pone.0043746 (PMC3423377; doi:10.1371/journal.pone.0043746)

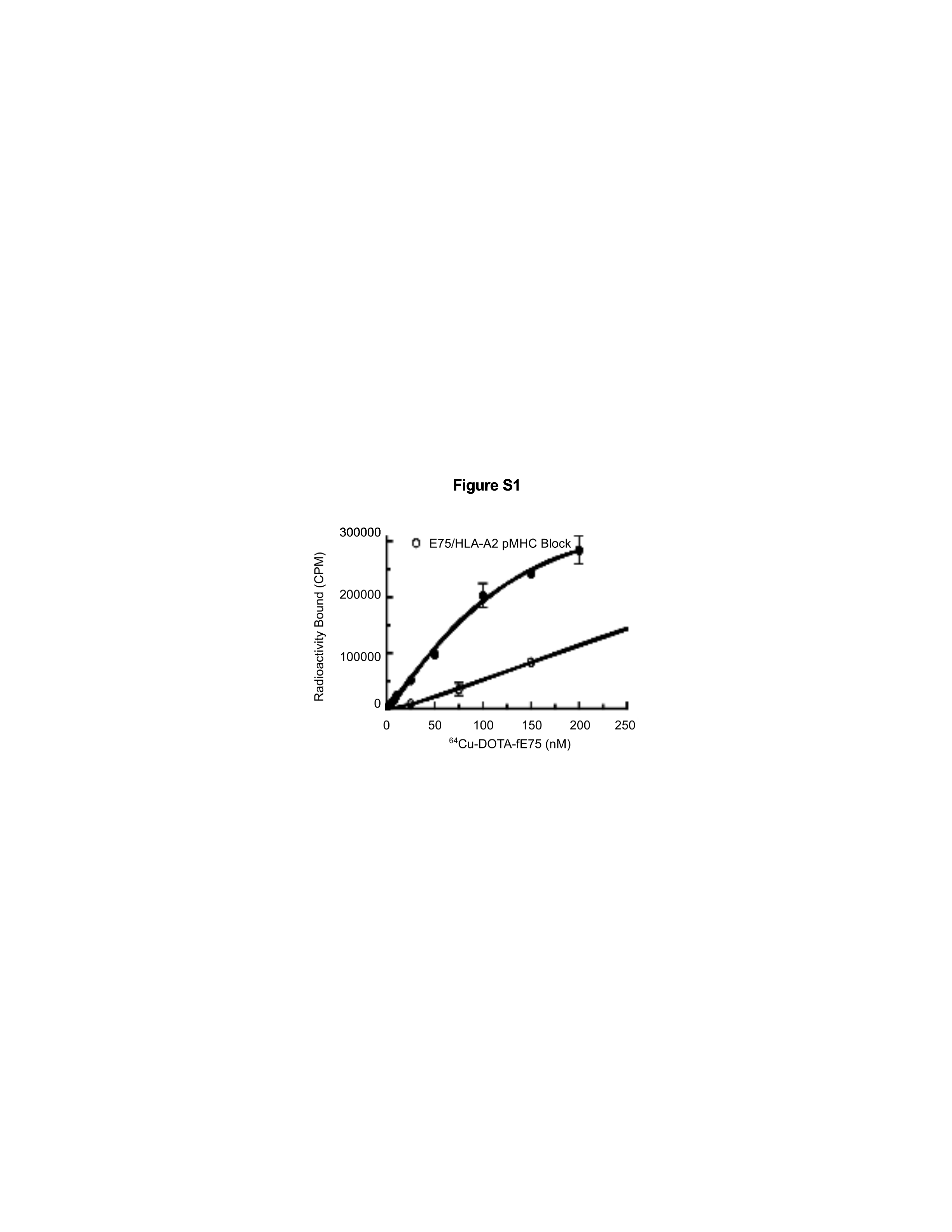

Supplement: Figure S1 — Saturation (•) binding curves of 64Cu-DOTA-fE75 and SKOV3 HLA-A2 (E75/HLA-A2 pMHC positive) cells. Each data point represents the mean ± standard error of the mean of triplicate measurements. The KD of 64Cu-DOTA-fE75 was determined to be 111 nM (95% CIs: 63–159). 64Cu-DOTA-fE75 binding (Ο) to SKOV3 HLA-A2 cells blocked by 300 nM soluble E75/HLA-A2 pMHC. (TIF) [file pone.0043746.s001.tif]

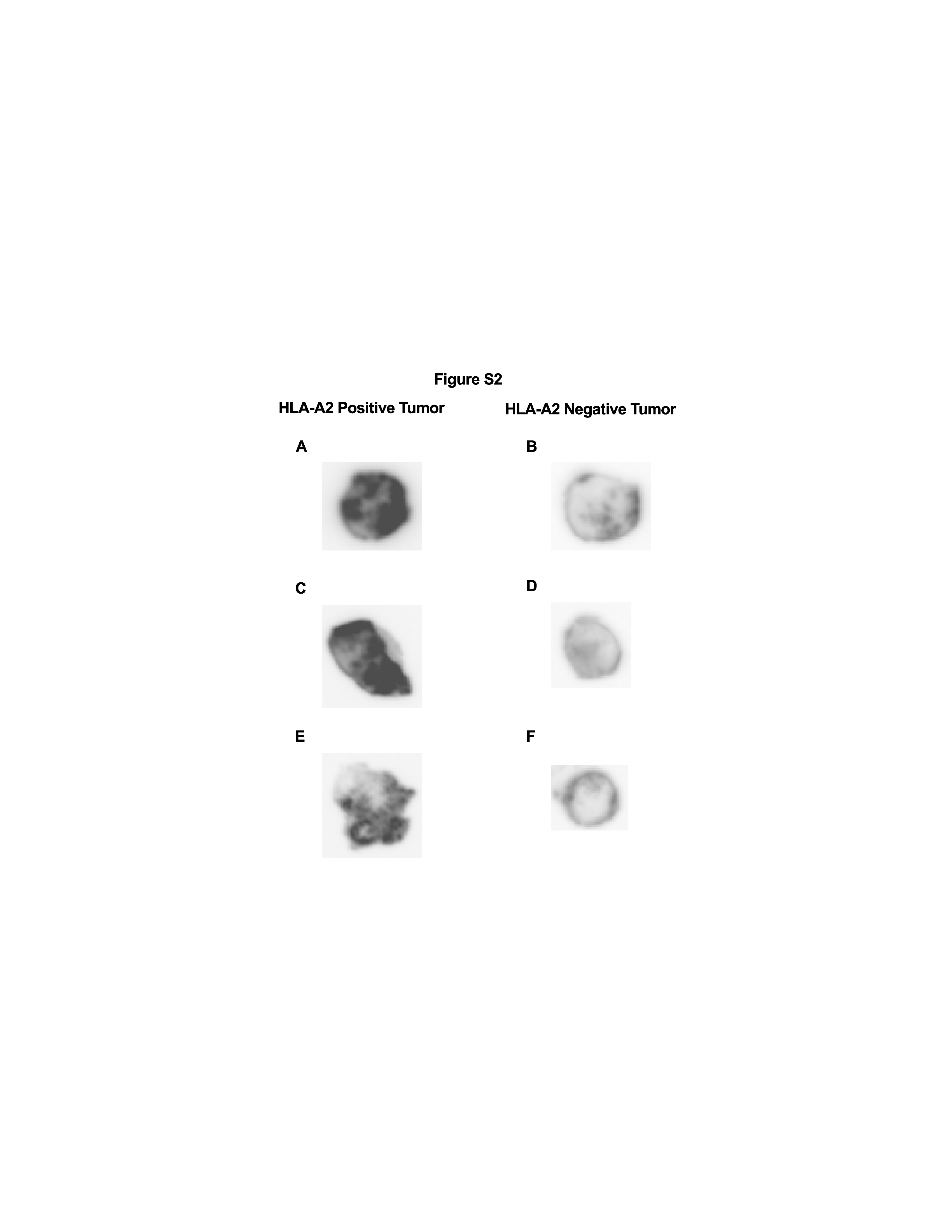

Supplement: Figure S2 — Radiography images of excised human tumors from 64Cu-DOTA-fE75 injected SCID and HLA-A2 trangenic SCID mice. HLA-A2 positive and HLA-A2 negative tumors from SCID mice were shown in A and B. HLA-A2 positive and HLA-A2 negative tumors from HLA-A2 transgenic SCID mice were shown in C–F. (TIF) [file pone.0043746.s002.tif]

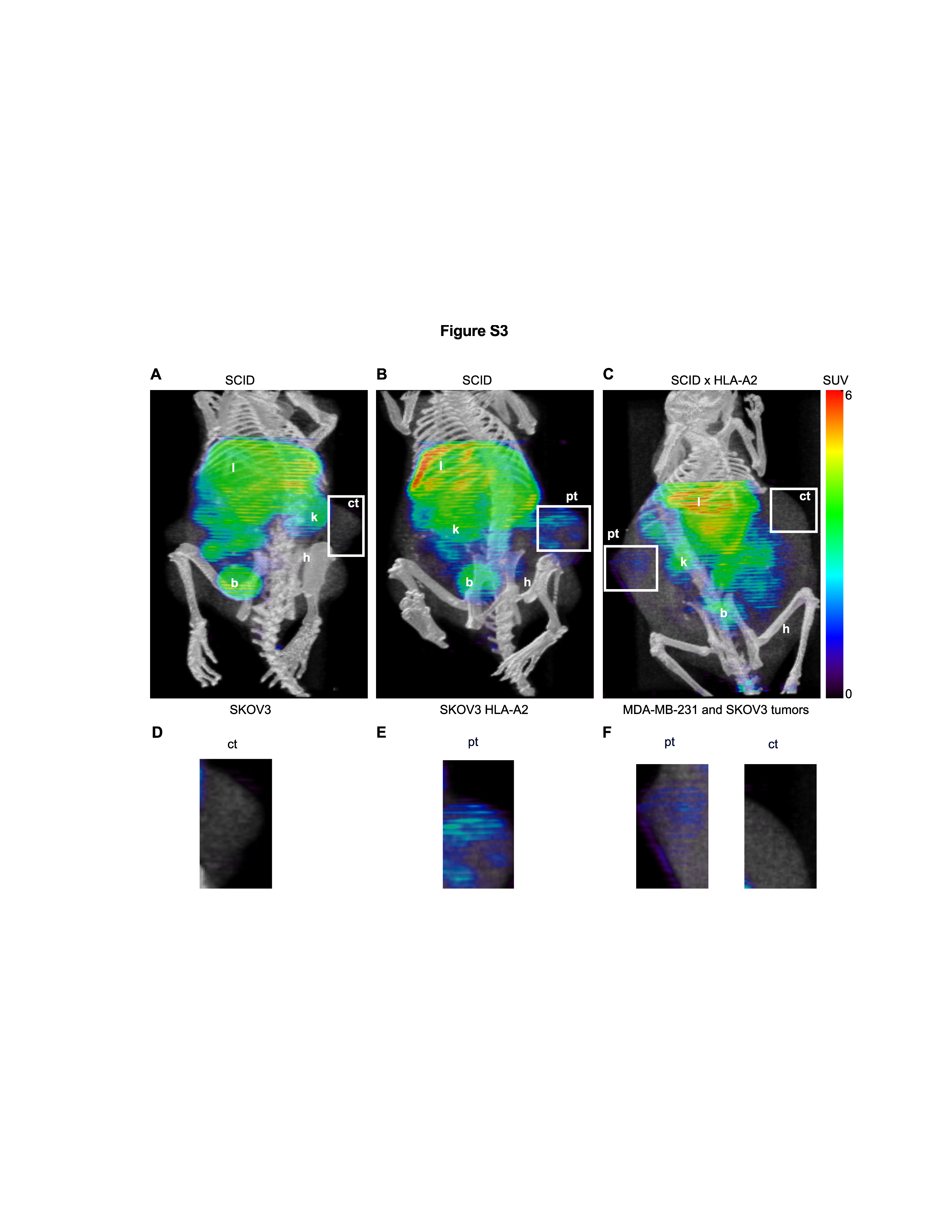

Supplement: Figure S3 — Additional PET/CT images of SCID and HLA-A2 transgenic SCID mice. Mice were injected intravenously with 64Cu-DOTA-fE75, which binds to tumors expressing both HLA-A2 and HER2/neu in vivo. Coronal views of small-animal PET images with coregistered CT images of SCID mice bearing human tumor (SKOV3 or SKOV3 HLA-A2) xenografts (A and B) or HLA-A2 transgenic SCID mouse bearing two tumors (SKOV3 and MDA-MB-231) xenografts (C) in flanks at 1 h post-injection were shown. Intensities of PET slices were scaled to the same maximum. Cropped views of only the tumors designated by the white boxes for the mice presented in A, B, and C were shown in D, E and F respectively. B = bladder, h = humeral muscle, k = kidneys, l = liver, ct = control HLA-A2 negative tumor; SKOV3 cell line, pt = HLA-A2 positive tumor; SKOV3 HLA-A2 or MDA-MB-231. (TIF) [file pone.0043746.s003.tif]
